# Urine sodium concentration to predict fluid responsiveness in oliguric ICU patients: a prospective multicenter observational study

**DOI:** 10.1186/s13054-016-1343-0

**Published:** 2016-05-29

**Authors:** Matthieu Legrand, Brigitte Le Cam, Sébastien Perbet, Claire Roger, Michael Darmon, Philippe Guerci, Axelle Ferry, Véronique Maurel, Sabri Soussi, Jean-Michel Constantin, Etienne Gayat, Jean-Yves Lefrant, Marc Leone, with the support of the AZUREA network

**Affiliations:** AP-HP, GH St-Louis-Lariboisière, Anesthésie, Réanimation et brûlés, Hôpital St-Louis, Assistance Publique-Hôpitaux de Paris, 1 rue Claude Vellefaux, 75010 Paris, France; UMR INSERM 942, Institut National de la Santé et de la Recherche Médicale (INSERM), Lariboisière Hospital, Paris, France; Université Paris Diderot, F-75475 Paris, France; Service de Réanimation Adultes et USC, CHU Estaing, Clermont-Ferrand, France; Service des Réanimations, Pôle Anesthésie Réanimation Douleur Urgence, CHU Nîmes, Place du Professeur Robert Debré, 30029 Nîmes Cedex 9, France; EA 2992, Faculté de Médecine, Université Montpellier, Nîmes, France; Service de Réanimation Médicale, Hôpital Nord, CHU Saint-Etienne, EA3065, Faculté de Médecine Jacques Lisfranc, Saint-Etienne, France; Département d’Anesthésie-Réanimation, CHU Nancy, Vandoeuvre-Lès-Nancy, France; Service d’Anesthésie-Réanimation, Hôpital Nord, AP-HM, Aix-Marseille Université, Marseille, France

**Keywords:** Fluid responsiveness, Natriuresis, Urine output, Acute kidney injury, Cardiac output

## Abstract

**Background:**

Oliguria is one of the leading triggers of fluid loading in patients in the intensive care unit (ICU). The purpose of this study was to assess the predictive value of urine Na^+^ (uNa^+^) and other routine urine biomarkers for cardiac fluid responsiveness in oliguric ICU patients.

**Methods:**

We conducted a prospective multicenter observational study in five university ICUs. Patients with urine output (UO) <0.5 ml/kg/h for 3 consecutive hours with a mean arterial pressure >65 mmHg received a fluid challenge. Cardiac fluid responsiveness was defined by an increase in stroke volume >15 % after fluid challenge. Urine and plasma biochemistry samples were examined before fluid challenge. We examined renal fluid responsiveness (defined as UO >0.5 ml/kg/h for 3 consecutive hours) after fluid challenge as a secondary endpoint.

**Results:**

Fifty-four patients (age 51 ± 37 years, Simplified Acute Physiology Score II score 40 ± 20) were included. Most patients (72 %) were not cardiac responders (CRs), and 50 % were renal responders (RRs) to fluid challenge. Patient characteristics were similar between CRs and cardiac nonresponders. uNa^+^ (37 ± 38 mmol/L vs 25 ± 75 mmol/L, *p* = 0.44) and fractional excretion of sodium (FENa^+^) (2.27 ± 2.5 % vs 2.15 ± 5.0 %, *p* = 0.94) were not statistically different between those who did and those who did not respond to the fluid challenge. Areas under the receiver operating characteristic (AUROC) curves were 0.51 (95 % CI 0.35–0.68) and 0.56 (95 % CI 0.39–0.73) for uNa^+^ and FENa^+^, respectively. Fractional excretion of urea had an AUROC curve of 0.70 (95 % CI 0.54–0.86, *p* = 0.03) for CRs. Baseline UO was higher in RRs than in renal nonresponders (1.07 ± 0.78 ml/kg/3 h vs 0.65 ± 0.53 ml/kg/3 h, *p* = 0.01). The AUROC curve for RRs was 0.65 (95 % CI 0.53–0.78) for uNa^+^.

**Conclusions:**

In the present study, most oliguric patients were not CRs and half were not renal responders to fluid challenge. Routine urinary biomarkers were not predictive of fluid responsiveness in oliguric normotensive ICU patients.

## Background

In the intensive care unit (ICU), oliguria is one of the first variables leading to a fluid challenge [1]. Although several physiological parameters may predict fluid responsiveness (e.g., increase in stroke volume after fluid challenge), most cannot be applied in ICU patients because criteria for validity are not met [2]. In addition, measurement of cardiac output is infrequent. As a result, at the bedside, fluid responsiveness remains difficult to assess [3]. In routine practice, oliguria is one of the leading conditions triggering the decision to apply fluid challenge [1]. This probably reflects the belief that oliguria is an accurate marker of hypovolemia.

A positive cumulative fluid balance has been associated with poor outcome, especially in patients with acute kidney injury (AKI) [4]. Hence, there is still a need for markers to predict fluid responsiveness in order to avoid an overload of fluid loading. Low urine Na^+^ concentration (uNa^+^) has long been considered a biomarker of a low intravascular volume state [5]. However, many factors may lead to renin-angiotensin-aldosterone system (RAAS) activation or alteration of intrarenal hemodynamics affecting uNa^+^ independently of intravascular volume [6]. However, the predictive value of uNa^+^ has never been assessed in ICU patients. We therefore conducted this study to evaluate the value of uNa^+^ for cardiac fluid responsiveness in normotensive, oliguric ICU patients. We also examined the renal responsiveness (i.e., urine output) after a fluid challenge as a secondary endpoint.

## Methods

Because this study was observational and did not change daily practice, the institutional review board of the University Hospital of Paris North (IRB00003835) approved the present study (protocol 2013/47NICB) and waived the requirement for obtaining written consent. However, the patients’ next of kin were systematically orally informed and could refuse patient participation. Moreover, the patients were later, and as soon as possible, systematically informed and could refuse use of their data.

### Patient selection

This study was performed in five university hospital ICUs. Patients were included if they met the following criteria:

1. They met criteria for oliguria, defined by urine output <0.5 ml/kg/h for 3 consecutive hours [7].
2. The administration of a fluid challenge (500 ml of isotonic crystalloids over 15 minutes) was indicated by the physician in charge.
3. Their mean arterial pressure was >65 mmHg and without significant change in norepinephrine dose (<20 %) during the last 3 h.
4. They did not receive diuretics on the day of inclusion.

Physicians were asked to explain the indications for fluid challenge. The exclusion criteria were patients under 18 years old, patients treated with diuretics, patients with stage 3 AKI, pregnancy, patients in whom a decision to withhold or withdraw treatment had been made, moribund patients, and patients who refused to participate.

### Measurements

The main endpoint of the study was fluid responsiveness, defined as an increase in stroke volume >15 % at the end of the fluid challenge. Patients were classified as cardiac responders (CRs) or cardiac nonresponders (CNRs) accordingly. Secondary endpoints included renal fluid responsiveness, defined as a post-fluid challenge urine output >0.5 ml/kg/h for more than 3 h (fluid renal responders [RRs]). Patient characteristics, Simplified Acute Physiology Score II (SAPS II) at admission, and reasons for ICU admission were recorded. At inclusion (i.e., at the time of oliguria diagnosis; urine output <0.5 ml/kg/h for 3 consecutive hours), we collected blood and urine samples, performed routine laboratory measurements, and recorded urine output during the previous 3 and 6 h.

Baseline serum creatinine (Screat) level was determined from blood samples taken on admission. In cases where the baseline creatinine plasma level or estimated glomerular filtration rate (eGFR) was not available, the baseline creatinine plasma level was estimated by using the Modification of Diet in Renal Disease equation with a normal eGFR value of 75 ml/minute/1.73 m^2^. New onset of AKI was defined as (1) an increase in Screat level 26.5 mol/L within 48 h or increase in SCreat to 1.5 times baseline ≥26 μmol/L or >50 % compared with baseline value or (2) need for renal replacement therapy (RRT) [8].

### Blood and urine analysis

Urine samples were immediately analyzed in the central laboratory of each participating center for uNa^+^, urine creatinine concentration, urine urea concentration (uUrea), and potassium concentration. Screat, Na^+^, potassium, urea, and chloride were measured at the same time, allowing calculation of fractional excretion of sodium (FENa^+^), fractional excretion of urea (FEurea), and urine/plasma creatinine.

### Statistical analysis

Using fluid responsiveness as the main endpoint, we estimated that an area under the receiver operating characteristic (AUROC) curve of uNa^+^ would be clinically relevant if the 95 % CI of its AUROC curve was >0.75, corresponding to a good AUROC [9, 10]. Inclusion of ≥40 patients was therefore necessary to show an AUROC curve of uNa^+^ >0.85 with 95 % CI >0.75 based on an estimation of 50 % of patients being fluid responders.

Categorical variables were compared using a χ^2^ test. The marginal association of biomarkers with fluid responsiveness was studied using the Mann-Whitney *U* test. The AUROC curve to predict fluid responsiveness was built for urine biochemistry biomarkers. We determined the optimal threshold value using the “closest top left” method. All analyses were performed using IBM SPSS Statistics software (IBM, Armonk, NY, USA). All *p* values were two-tailed, and a *p* value <0.05 was considered significant. Values are expressed as number and percentage or median and interquartile range accordingly.

## Results

### Patient selection

Fifty-four patients were included (age 64 ± 19 years, *n* = 22 females, SAPS II score 38 ± 17) between March 2014 and March 2015. The patient features are shown in Table 1. Reasons for ICU admission were sepsis, neurological disorders, acute respiratory failure, and trauma/burns. At inclusion, seven patients (13 %) were being treated with antibiotics.

**Table 1:** Patient characteristics

Characteristic	All patients (*n* = 54)	Cardiac responders (*n* = 15)	Cardiac nonresponders (*n* = 39)	*p* Value
Age, years	64 (55–73)	66 (54– 78)	63 (54–72)	0.93
Male sex, *n* (%)	32 (59)	8 (53)	23 (59)	0.73
Comorbidities, *n* (%)				
COPD	8 (15)	3 (20)	5 (13)	0.67
Diabetes mellitus	8 (15)	2 (13)	6 (15)	1.0
Hypertension	23 (42)	7 (47)	16 (41)	0.76
Heart failure	8 (15)	5 (33)	3 (8)	0.03
Cancer	11 (20)	4 (27)	7 (18)	0.47
Nephrotoxic agents				
NSAIDs	1 (2)	0 (0)	1 (3)	1.0
ACE inhibitors	2 (4)	0 (0)	2 (5)	1.0
Aminoglycosides	9 (17)	2 (13)	7 (18)	1.0
Contrast media	12 (22)	16 (23)	9 (23)	1.0
Organ failure				
Mechanical ventilation, *n* (%)	33 (42)	10 (67)	23 (59)	0.77
SAPS II score	39 (30–48)	38 (25–51)	39 (30–48)	0.92
Norepinephrine, *n* (%)	17 (31)	6 (40)	11 (28)	0.52
Lactate, mmol/L	1.8 (1.3–2.3)	1.9 (0.9–2.8)	1.7 (1.3–2.2)	0.39
Blood urea nitrogen, mmol/L	12 (8–17)	11 (7–15)	13 (8–18)	0.93
Serum creatinine, μmol/L	90 (56–124)	117 (81–153)	74 (49–99)	0.22
Bilirubin, mg/L	7 (1–14)	9 (5–13)	7 (1–13)	1.00
Bicarbonate, mmol/L	25 (22–28)	21 (17–25)	26 (23–29)	0.06
Platelet count, 10^3^/L	346 (201–491)	207 (95–319)	358 (215–501)	1.00
Hemoglobin, g/dl	9.4 (8.1–10.7)	10.4 (8.4–12.4)	9.1 (8.1–10.1)	0.54
Glucose, mmol/L	7.9 (7.0–8.8)	7.9 (7.1–8.6)	8.0 (6.9–9.1)	0.73
Reason for ICU admission				
Sepsis	14 (26)	2 (13)	12 (31)	0.3
Neuro-ICU	5 (9)	2 (13)	3 (8)	0.61
Respiratory failure	13 (24)	1 (7)	12 (31)	0.08
Trauma/hemorrhage	7 (13)	2 (13)	5 (13)	1.0
Cardiogenic shock	1 (2)	0 (0)	1 (3)	1.0
Post-cardiac arrest	4 (7)	2 (13)	2 (5)	0.3
Hemodynamic status				
SAP, mmHg	134 (118–150)	112 (104–120)	138 (126–150)	0.92
MAP, mmHg	81 (71–91)	74 (67–81)	87 (79–95)	0.07
HR, beats/minute	98 (85–111)	98 (82–114)	96 (81–111)	0.14
CO, L/minute	5.3 (2.8–7.8)	4.9 (1.7–8.1)	5.6 (3.3–7.9)	0.07
ScvO_2_, %	75 (62–88)	66 (54–78)	85 (75–95)	0.01
CVP, mmHg	8 (6–10)	8 (7–9)	7 (4–10)	0.58

*Abbreviations: ICU* intensive care unit, *COPD* chronic obstructive pulmonary disease, *CAD* coronary artery disease, *NSAIDs* nonsteroidal anti-inflammatory drugs, *ACE* angiotensin-converting enzyme, *SAPS II* Simplified Acute Physiology Score II, *SAP* systolic arterial pressure, *MAP* mean arterial pressure, *HR* heart rate, *CO* cardiac output, *CVP* central venous pressure, *ScvO*_*2*_ central venous oxygen saturation

Data are expressed as absolute number (percentage) or median (interquartile range)

### Fluid challenge

Stroke volume was measured using calibrated pulse pressure signal analysis (*n* = 13), ultrasound-derived analysis (transesophageal Doppler; *n* = 17), or echocardiography (*n* = 24). Before fluid challenge, only cardiac output and central venous oxygen saturation were lower in the CRs than in the CNRs (Table 1). Pulse pressure variations were similar in the CRs and the CNRs (14 ± 19 % vs 6 ± 11 %, *p* = 0.18). During the inclusion day, the CRs and the CNRs received 1543 ± 1415 ml and 2253 ± 2381 ml of fluid, respectively (*p* = 0.28).

### Biomarkers to predict fluid responsiveness

#### Cardiac response

**Fig. 1:**
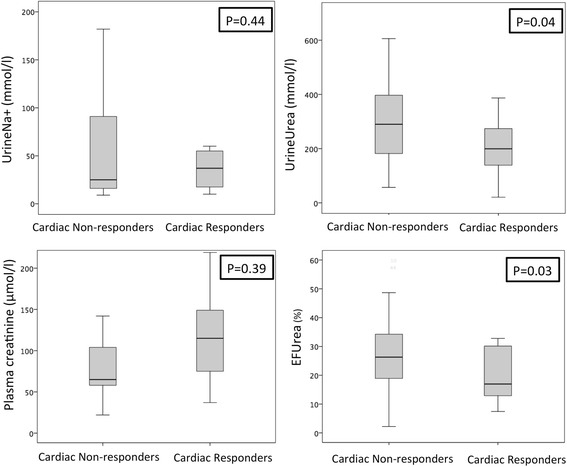
Box plots representing urine Na^+^, serum creatinine, urine urea, and fractional excretion of urea (FEurea) at the time of oliguria recognition, according to cardiac fluid responsiveness

**Fig. 2:**
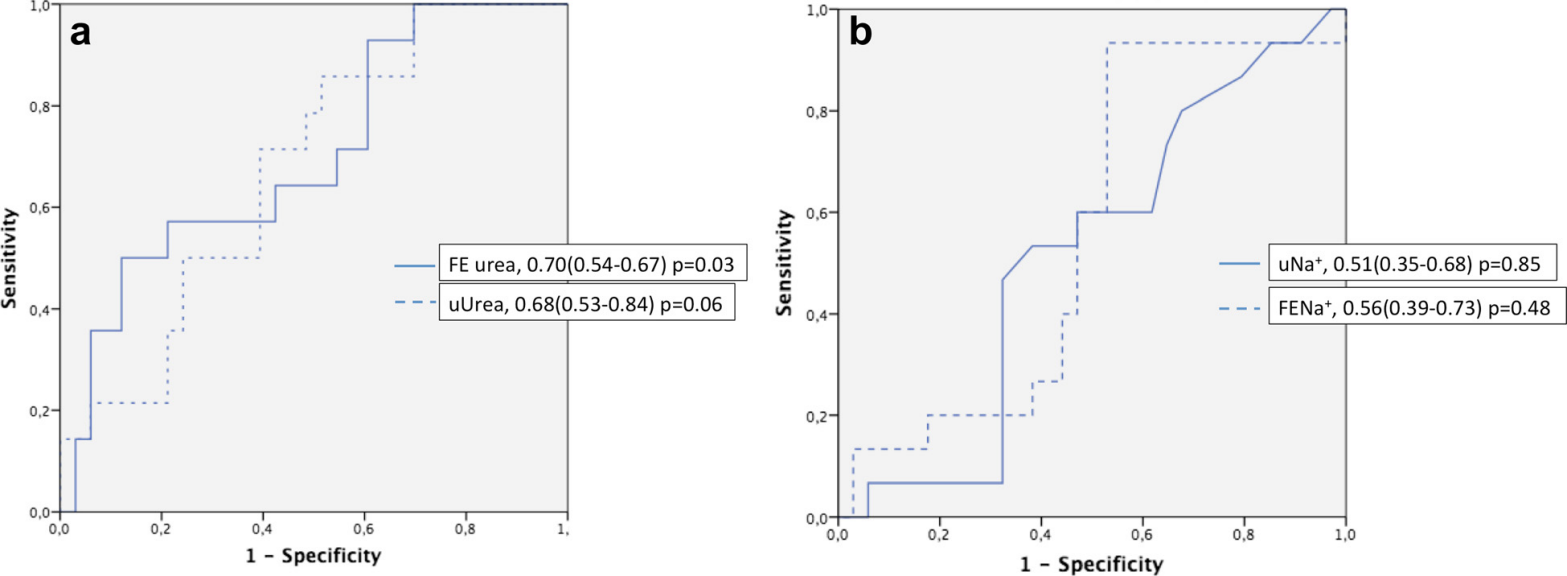
**a** Receiver operating characteristic curves of urine urea (uUrea) and fractional excretion of urea (FEurea). **b** Receiver operating characteristic curves of urine Na ^+^ (uNa^+^) and fractional excretion of Na ^+^ (FENa^+^) at the time of oliguria recognition to predict cardiac fluid responsiveness. Data are expressed as median (95 % CI)

Fifteen patients (27 %) were CRs to the fluid challenge. Baseline urine output (0.76 ± 0.90 ml/kg/3 h vs 0.94 ± 0.69 ml/kg/3 h, *p* = 0.55), Urine soidium ( uNa^+^ 37 ± 38 mmol/L vs 25 ± 75 mmol/L, *p* = 0.88), and FENa^+^ (2.3 ± 2.5 % vs 2.2 ± 5.0 %, *p* = 0.40) were similar in the CRs and the CNRs, corresponding to AUROC curves for predicting fluid responsiveness of 0.51 (95 % CI 0.35–0.68) and 0.56 (95 % CI 0.39-0.73), respectively, for uNa^i^ and FENa^+^ (Fig. 1). uNa^+^ <20 mmol/L and FENa^+^ <1 % had sensitivities of 40 % and 93 %, respectively, and specificities of 61 % and 41 %, respectively, to predict the cardiac response. FEurea (17 ± 17 % vs 26 ± 16 %, *p* = 0.036) and uUrea (200 ± 154 mmol/L vs 299 ± 214 mmol/L, *p* = 0.04) were less in the CRs than in the CNRs (Fig. 2), corresponding to AUROC curves of 0.70 (95 % CI 0.54–0.86, *p* = 0.03) and 0.68 (95 % CI 0.53–0.84, *p* = 0.06), respectively (Fig. 2).

#### Renal response

**Fig. 3:**
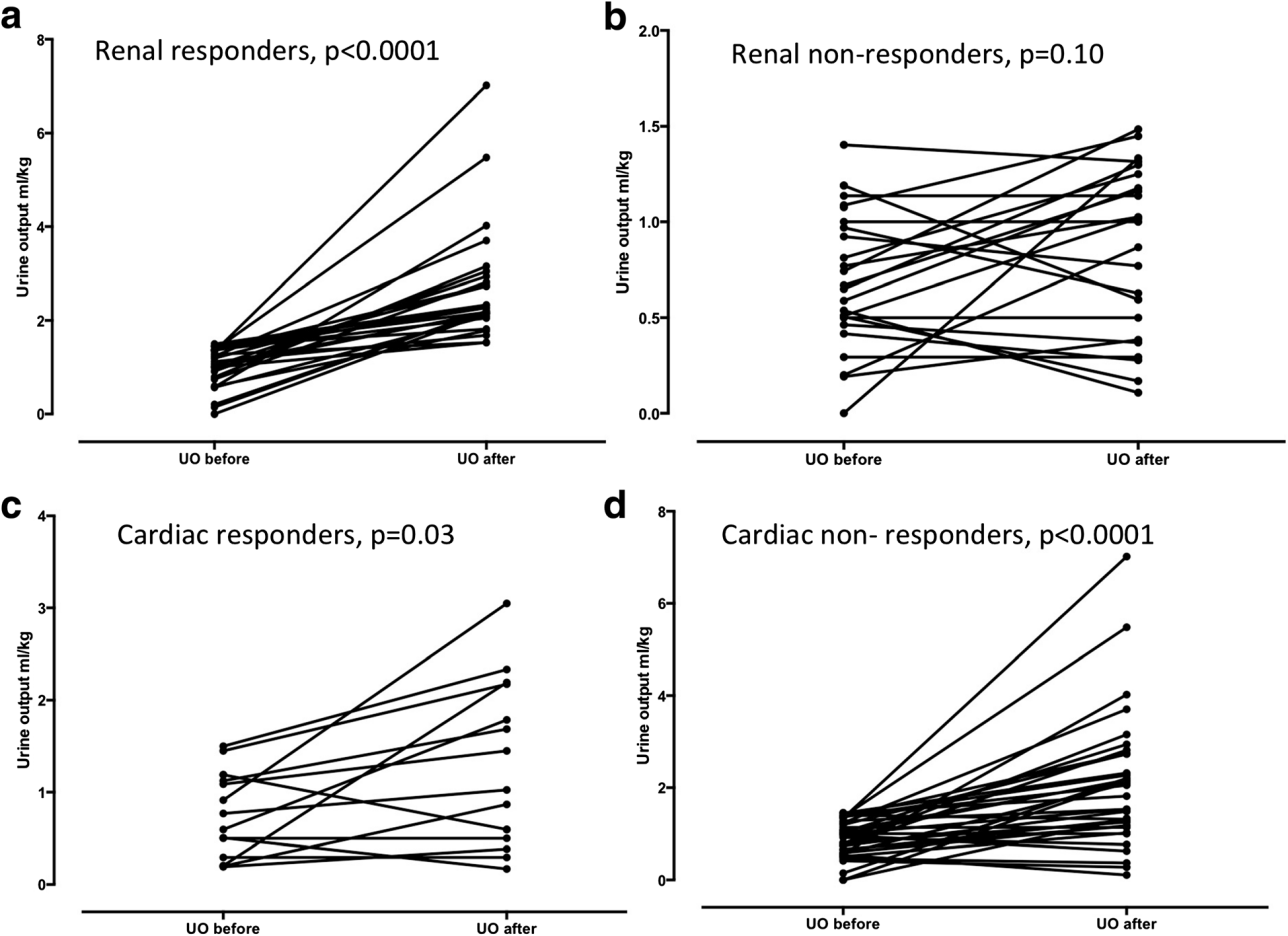
Evolution of urine output (UO) between 3 h before and after fluid challenge, respectively, in renal responders (**a**), renal nonresponders (**b**), cardiac responders (**c**), and cardiac nonresponders (**d**)

Twenty-seven patients (50 %) were RRs to the fluid challenge. These changes persisted 6 h after the fluid challenge. Baseline urine output was 1.07 ± 0.78 ml/kg/3 h in the RRs and 0.65 ± 0.53 ml/kg/3 h in the renal nonresponders (*p* = 0.01). The AUROC curves for predicting renal fluid responsiveness were 0.65 (95 % CI 0.53–0.78) for uNa^+^, 0.57 (95 % CI 0.41–0.73) for FENa^+^, and 0.61 (95 % CI 0.45–0.77) for FEUrea. Urine output increased to 1.03 ± 1.67 ml/kg/3 h in CRs and to 1.81 ± 1.38 ml/kg/3 h in CNRs (*p* = 0.03 and *p* < 0.001, respectively) compared with baseline during the 3 h after the fluid challenge (Fig. 3).

### Outcome

Twenty-one patients developed AKI, including seven patients (13 %) requiring RRT (*p* > 0.05 between fluid responders and fluid nonresponders). Eleven patients (21 %) died during their ICU stay (*p* > 0.05 between CRs and CNRs).

## Discussion

In our study, most patients with transient oliguria did not increase their cardiac output or their urine output after the fluid challenge. Low urine sodium concentration and FENa^+^ were not reliable predictors of fluid responsiveness. Although FEurea and uUrea were different in the CRs and the CNRs, the differences were not clinically relevant for predicting fluid responsiveness.

In routine practice, low urine output often leads to performing fluid challenge in ICU patients. This is based on the hypothesis of a systemic hemodynamic contribution to low renal blood flow and low urine output [11, 12]. However, physiological grounds exist to consider oliguria a poor marker of hypovolemia or low intravascular volume [6]. Shock, pain, and the perioperative period are associated with alteration of intrarenal hemodynamics [13, 14] and activation of the RAAS, leading to antinatriuresis and antidiuresis [15, 16]. Our study shows that oliguria with low urine Na^+^ concentration in normotensive ICU patients may not reflect hypovolemia in a large proportion of patients. Hence, fluid challenge may not translate into an increase in cardiac output or urine output.

Low uNa^+^ has long been proposed as a biomarker of prerenal failure as well as low intravascular volume status and/or low cardiac output. However, a low urine Na^+^ concentration is a biomarker of RAAS activation that may be triggered by various factors. Urine biomarkers have previously been shown to inaccurately predict persistent or transient AKI [17]. In a multicenter study, Pons et al. showed that urine biochemistry parameters, including FENa^+^ and FEurea, did not predict the rapid reversibility of AKI [18]. Regulation of urine output and renal Na^+^ handling involves many other factors, including tubular cell function and systemic inflammation [19, 20]. Therefore, many factors unrelated to intravascular volume and cardiac output may affect urine output and renal Na^+^ handling, including tubular Na^+^ channel expression [6]. It therefore remains very difficult to recommend the use of urinary biomarkers to predict fluid responsiveness in critically ill patients. Urine biomarkers may still be indicative of tubular function and renal hemodynamics, but these points were not addressed in the present study. In our study, the influence of renal perfusion pressure on renal hemodynamics was reduced by excluding the patients with mean arterial pressure <65 mmHg. However, urine Na^+^ remained poorly predictive of fluid responsiveness (either cardiac output or renal response) in our patients. Clinicians should therefore rely on other markers of fluid responsiveness when deciding whether to initiate a fluid challenge in patients with oliguria. Nevertheless, we acknowledge that our definition of renal responsiveness was arbitrary, relying on the Kidney Disease: Improving Global Outcomes (commonly referred to as KDIGO [21]) definition with a urine output threshold of 0.5 ml/kg/h. Some patients showed a relative increase of urine output after the fluid challenge, albeit below this threshold.

Our study has several limitations. First, the sample size was small. However, the sample size was sufficient to detect a good predictive value of the biomarker. This hypothesis can be rejected on the basis of our results. The low rate of inclusion could be explained by several factors. Our patients had to be both oliguric and hemodynamically stabilized, which may have limited the number of patients eligible for inclusion. Second, they had to be off drugs that interact with renal sodium handling (e.g., diuretics). Third, monitoring of cardiac output or an echocardiography-certified physician was required to monitor stroke volume during the fluid challenge. Altogether, these points may limit the external validity of the results. Nevertheless, urine Na^+^ was not strongly associated with fluid responsiveness. A poor predictive value for fluid responsiveness was observed.

Stroke volume was evaluated with different tools. These tools have been used with acceptable accuracy in monitoring stroke volume [2]. Most important, the same monitor was used for each patient, limiting bias. Urine biochemistry analyses were not centralized in a single laboratory. This may have generated variability, but it also increased the extrapolation of our results. Norepinephrine infusion was required in some patients, affecting intrarenal hemodynamics. However, Bellomo et al. observed that restoring renal perfusion pressure with norepinephrine decreased renal resistance and increased renal conductance in sepsis [22]. Finally, regarding the AKI definition, we were aware that estimating baseline serum creatinine may have introduced bias into the classification. However, estimation of baseline serum creatinine in ICU patients with no available baseline values remains a challenge, with no consensus on the method of obtaining a surrogate.

## Conclusions

In the present study, most oliguric patients were not cardiac responders and half were not renal responders to fluid challenge. Routine urinary biomarkers were not predictive of fluid responsiveness in oliguric, normotensive ICU patients.

## Key messages

- Most ICU normotensive patients with oliguria do not respond to fluid challenge.
- Urine Na ^+^ and urea concentration are not reliable biomarkers of fluid responsiveness in oliguric ICU patients.
- Excessive reliance on oliguria and urine biomarkers may lead to excessive fluid loading with potentially harmful consequences.

## Abbreviations

ACE: angiotensin-converting enzyme
AKI: acute kidney injury
CAD: coronary artery disease
CO: cardiac output
COPD: chronic obstructive pulmonary disease
CVP: central venous pressure
eGFR: estimated glomerular filtration rate
FENa^+^: fractional excretion of sodium
FEurea: fractional excretion of urea
HR: heart rate
ICU: intensive care unit
MAP: mean arterial pressure
NSAID: nonsteroidal anti-inflammatory drug
RAAS: renin-angiotensin-aldosterone system
RRT: renal replacement therapy
SAP: systolic arterial pressure
SAPS II: Simplified Acute Physiology Score II
Screat: serum creatinine
ScvO_2_: central venous oxygen saturation
uNa^+^: urine Na^+^ concentration
UO: urine output
uUrea: urine urea concentration
